# Patellar instability: the reliability of magnetic resonance imaging measurement parameters

**DOI:** 10.1186/s12891-019-2697-7

**Published:** 2019-07-06

**Authors:** Qin Ye, Taihen Yu, Yinbo Wu, Xiaonan Ding, Xiangyang Gong

**Affiliations:** 1 Zhejiang Chinese Medical University, Hangzhou, China; 2 Department of Radiology, Zhejiang Provincial People’s Hospital, Affiliated People’s Hospital of Hangzhou Medical College, Hangzhou, China

**Keywords:** Patellar instability, Reliability, Measurement, Magnetic resonance imaging

## Abstract

**Background:**

Radiological assessments are considered an important part of the management of patellar instability (PI). However, PI measurements are influenced by the knee position, which cannot be guaranteed to be the same for each examination. Therefore, we aimed to determine the reliability of common PI measurements on magnetic resonance imaging (MRI).

**Methods:**

Two MRI examinations within a 6-month period were obtained from 51 knees. The common PI measurements were quantitatively determined and re-evaluated. The intraclass correlation coefficients (ICC), Bland–Altman plot, standard error of measurement (SEM), and minimal detectable change (MDC) were used to determine the intra-observer, inter-observer, and inter-scan reliability.

**Results:**

Adequate intra- and inter-observer reliability was obtained for all PI measurements (all ICCs > 0.8). For patellar positional parameters, the inter-scan reliability was adequate for the angle of Fulkerson, angle of Laurin, patellar tilt angle (PTA), lateral patellar displacement (LPD), and bisect offset ratio (BSO; ICCs = 0.723–0.897), although it was inadequate for the angle of Grelsamer and the congruence angle (CA; ICCs = 0.325–0.380). All parameters of trochlear dysplasia showed adequate inter-scan reliability (ICCs = 0.793–0.915). Nearly all patellar height parameters showed adequate inter-scan reliability (ICCs = 0.700–0.903), except the patellar trochlear index (PTI; ICC = 0.655).

**Conclusion:**

All PI measurements showed adequate intra- and inter-observer reliability on MRI. Most measurements showed adequate inter-scan reliability, with the exception of the angle of Grelsamer, CA, and PTI.

**Electronic supplementary material:**

The online version of this article (10.1186/s12891-019-2697-7) contains supplementary material, which is available to authorized users.

## Background

The patellofemoral joint is stabilized by a complex multivariate relationship of osseous joint geometry and the force vectors produced by the quadriceps femoris and capsuloligamentous stabilizers [1]. Patellar instability (PI) refers to a clinical condition that is often caused by pathomorphologic changes that involve the patellofemoral joint stabilizers, thereby increasing the possibility of lateral patellar dislocation and early osteoarthritis [2]. The incidence of primary PI is 5.8 per 100,000 in the general population, with higher incidence in younger and more active individuals [3].

Several predisposing risk factors, such as trochlear dysplasia, patellar alta, insufficient medial patellofemoral ligament, and lateralization of the tibial tuberosity, are thought to contribute to PI [1, 2, 4]. Thus, the main purpose of radiological examinations is to confirm the diagnosis and determine the primary factors that contribute to PI. Magnetic resonance imaging (MRI) is a widely used imaging modality for knee disorders as well as for PI [5, 6]. MRI has been found to be highly sensitive in detecting capsular, ligament, cartilaginous, and bone injuries related to patellar dislocation [6–8].

Numerous MRI-based quantitative measurement parameters have been proposed as diagnostic criteria and/or guiding factors for clinical management strategies in cases of PI [4]. However, there are some clinical factors that may affect the reliability of these measurement parameters, such as the knee flexion angle and quadriceps action [9–13]. In clinical practice, the knee is usually flexed to some degree due to the MRI coil shape, the knee position in the coil, and differences in operator habits [12]. Between conventional MRI scans, it is hard to set a uniform examination position of the knee even for the same individual. The inter-scan reliability of MRI measurements for PI are yet to be evaluated. The aim of this study is to determine intra-observer, inter-observer, and inter-scan reliability of a series of established MRI quantitative measurement parameters for PI.

## Methods

### Participants

We retrospectively collected imaging and clinical data from July 2015 to May 2018 via the Picture Archiving and Communication System (PACS) at our institution. Patients were eligible for inclusion in the study if they had at least two MRI exams of the same knee conducted on the same MRI unit within 6 months. Because we were concerned with the reliability of the PI measurements rather than their validity, we included a variety of knee pathologies, which were not limited to patients with PI. Patients were excluded from this study if they had an: (1) acute disease of the knee, including ligament injury, massive effusion or osteonecrosis, and acute injury from a motor vehicle accident or fall; (2) previous knee surgery; and (3) poor MRI image quality.

### MRI technique

The MRIs were conducted using a 3.0-T MRI scanner (Discovery MR 750; GE Healthcare, Waukesha, WI, USA) with a dedicated 8-channel knee coil. The participants were placed in the supine position, with dedicated sponge pads above/under the knee joint to prevent motion during the examination. Axial and sagittal proton density-weighted fat-saturated images of the knee were used for this study. The acquisition parameters included: (1) sagittal images with a repetition time (TR) of 2846 ms, an echo time (TE) of 35 ms, 3-mm slice thickness, a field of view (FOV) of 16 × 16 cm, an echo train length of 12, a matrix of 352 × 224 pixels, two excitations, and a 114-s scan time; and (2) axial images with a TR of 2000 ms, a TE of 35 ms, 3-mm slice thickness, an FOV of 16 × 16 cm, an echo train length of 8, a matrix of 320 × 224 pixels, two excitations, and a 116-s scan time.

### Image evaluation

Quantitative measurements were undertaken on a PACS workstation (Greenland, version 6.0) with a dedicated monitor (Jusha, M11 M21 C21). Two senior musculoskeletal radiologists (with 20 and 16 years of experience, respectively) independently measured all parameters in the participants. The reliability between the measurements of the two reviewers was defined as the inter-observer reliability. After a minimum interval of 6 weeks, each MRI assessment was re-evaluated by the one of these two reviewers. The reliability between two measurements of the same reviewer was defined as the intra-observer reliability. The reliability of values of the most senior radiologist among two reviewers between two MRI scans was defined as the inter-scan reliability.

The parameters that were measured by superimposing image slices in the present study included the: (1) angle of Fulkerson [9], angle of Grelsamer [9], angle of Laurin [9], patellar tilt angle (PTA) [9, 10, 14], lateral patellar displacement (LPD) [15], bisect offset ratio (BSO) [10], and congruence angle (CA) [9] for patellar position; (2) sulcus angle (SA) [9, 14], lateral trochlear inclination (LTI) [14], and trochlear facet asymmetry (TFA) [14], and trochlear groove depth (TGD) [14, 16] for trochlear dysplasia; and (3) Insall–Salvati index (ISI) [9, 10, 14], modified Insall–Salvati index (MISI) [9], Caton–Deschamps index (CDI), Blackburne–Peel index (BPI) [13], and patellar trochlear index (PTI) [10, 14] for patellar height (Additional file 1, Figs. 1, 2 and 3). The angle between the femoral shaft and the tibial shaft in MR images was defined as the flexion angle [11].

**Fig. 1:**
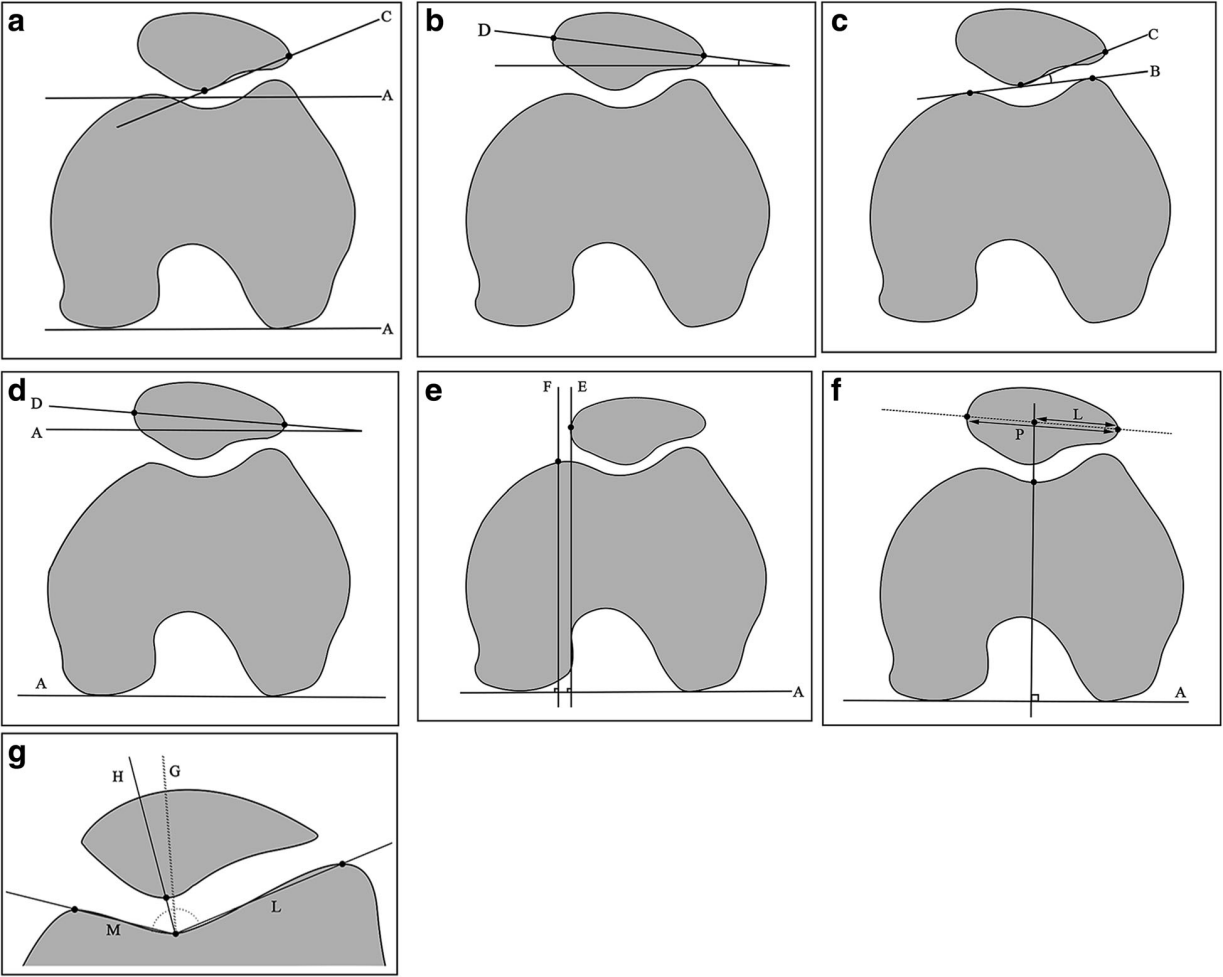
Measurements of patellar position: **a** the angle of Fulkerson is the angle between C and A; **b** the angle of Grelsamer is the angle between D and the horizontal axis; **c** the angle of Laurin is the angle between C and B; **d** patellar tilt angle (PTA) is the angle between D and A; **e** lateral patellar displacement (LPD) is the shortest distance between E and F; **f** bisect offset ratio (BSO) = L/P; and (**g**) congruence angle (CA) is the angle between G and H

**Fig. 2:**
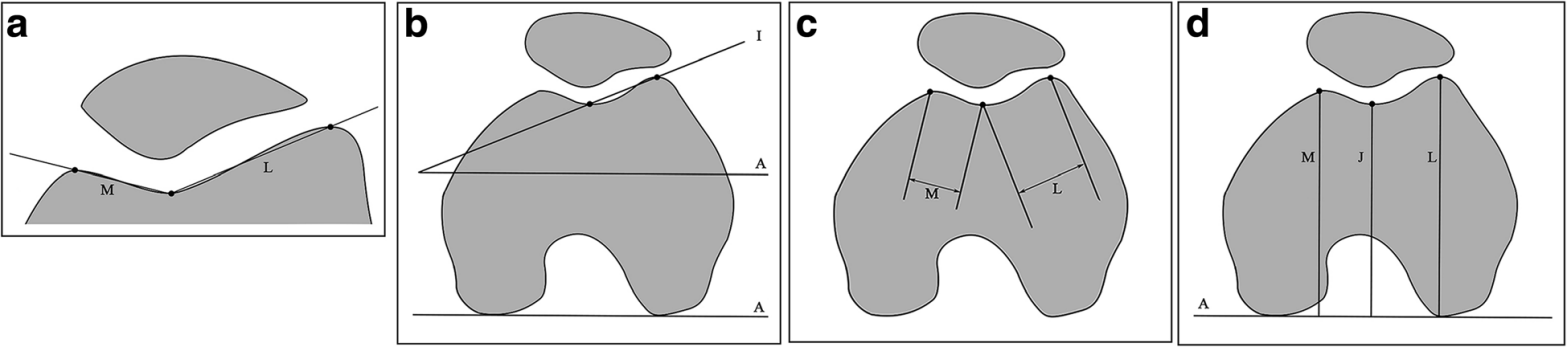
Measurements for trochlear dysplasia: **a** sulcus angle (SA) is the angle between M and L; **b** lateral trochlear inclination (LTI) is the angle between I and A; **c** trochlear facet asymmetry (TFA) = M/L; and (**d**) trochlear groove depth (TGD) = (M + L)/2 − J

**Fig. 3:**
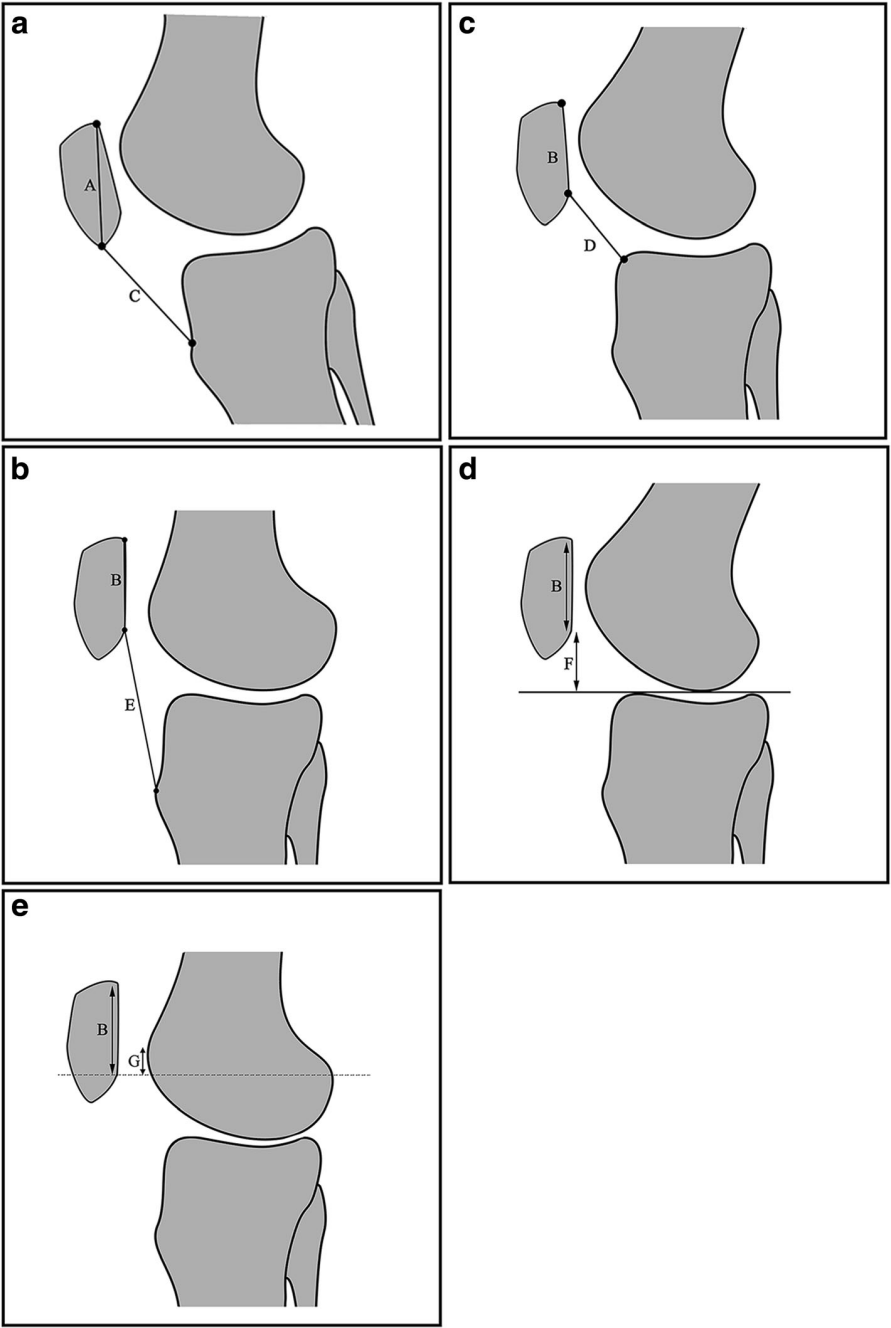
Measurements for patellar height: **a** Insall–Salvati index (ISI) = C/A; **b** modified Insall–Salvati index (MISI) = E/B; **c** Caton–Deschamps index (CDI) = D/B; **d** Blackburne–Peel index (BPI) = F/B; and (**e**) patellar trochlear index (PTI) = G/B

### Statistical analysis

Statistical analysis was carried out the Medcalc 16.2 software and R version 3.3.3 (R Foundation for Statistical Computing, Vienna, Austria). Demographic factors, including age, sex, body mass index (BMI), affected side, diagnosis, and knee flexion angle, of the participants were computed by descriptive statistics. Intra-observer, inter-observer, and inter-scan reliability were evaluated using the intra-class correlation coefficient (ICC), Bland–Altman plot, standard error of measurement (SEM) and minimal detectable change at 95% confidence level (MDC_95_) for continuous quantitative variables.

For intra-observer, inter-observer, and inter-scan reliability, we used ICC (3,1), ICC (2,1), and ICC (2,1), respectively, with the definition of absolute agreement [17]. Values greater than or equal to 0.70 were considered adequate for reliability [18].

The measurement error was assessed visually using the Bland–Altman plot, as determined by the 95% limits of agreement (LoA) and the mean difference [19].

In addition, we used SEM to evaluate the measurement error. The MDC_95_ was used to determine whether a true change had taken place beyond the measurement error. The SEM and MDC_95_ were calculated with the following formulas: SEM = SD × √ (1 - r) (SD: standard deviation, r: coefficient of the reliability) and MDC_95_ = 1.96 × √2 × SEM [20].

## Results

### Demographic characteristics

Initially, we included 316 patients, of whom 265 patients were excluded because of acute knee disease (*n* = 145), previous knee surgery (*n* = 116), and poor image quality (*n* = 4). The remaining 51 patients (32 males and 19 females; mean age: 33.78 ± 9.63 years) were enrolled into the study. The demographic characteristics of the study population are summarized in Table 1. The mean difference of the knee flexion angle between two scans of the same patient was 3.57°.

**Table 1:** Demographic characteristics of the participants (*N* = 51)

Characteristics	Value
Sex, no. (%)	
Male	32 (63)
Female	19 (37)
Affected knee, no. (%)	
Right	24 (47)
Left	27 (53)
Diagnosis, no. (%)	
Patellar instability	2 (4)
Meniscal injury	16 (31)
Knee pain without injury	33 (65)
Knee flexion angle, deg., mean ± SD	7.25 ± 4.68
Age, y, mean ± SD	33.78 ± 9.63
Body mass index, mean ± SD	22.25 ± 2.78

*SD* Standard deviation, *deg* degree, *y* year

### Intra-observer reliability

We found adequate intra-observer reliability for all parameters that describe patellar position, trochlear dysplasia, and patellar height (ICCs = 0.851–0.980). The 95% LoA for the CA between the two evaluations was slightly wider than those for the other MRI measurements. The detailed intra-observer reliability is presented in Table 2.

**Table 2:** Intra-observer reliability of the MRI measurements for patellar instability

Measurement	ICC	95%CI	Mean difference	95%LoA	SEM	MDC_95_
Patellar position						
Angle of Fulkerson, deg	0.969	0.953~0.979	0.216	−2.019~2.450	0.808	2.240
Angle of Grelsamer, deg	0.938	0.910~0.958	0.000	− 3.096~3.096	1.111	3.080
Angle of Laurin, deg	0.945	0.919~0.962	−0.039	−3.325~3.247	1.175	3.257
PTA, deg	0.953	0.931~0.968	0.069	−2.276~2.413	0.841	2.330
LPD, mm	0.980	0.970~0.987	−0.085	− 0.899~0.728	0.298	0.825
BSO, %	0.898	0.852~0.930	−0.005	− 0.047~0.038	0.015	0.043
CA, deg	0.908	0.866~0.937	−0.892	−11.037~9.253	3.685	10.215
Trochlear dysplasia						
SA, deg	0.964	0.947~0.975	0.098	−3.798~3.994	1.390	3.852
LTI, deg	0.940	0.912~0.960	−0.324	−3.152~2.505	1.040	2.883
TFA, %	0.881	0.830~0.918	0.008	−0.103~0.119	0.040	0.112
TGD, mm	0.940	0.913~0.959	−0.012	−0.937~0.912	0.331	0.919
Patellar height						
ISI	0.876	0.807~0.922	−0.009	− 0.128~0.111	0.043	0.119
MISI	0.911	0.859~0.944	−0.018	−0.187~0.152	0.062	0.171
CDI	0.917	0.869~0.948	−0.007	− 0.107~0.092	0.036	0.100
BPI	0.851	0.769~0.905	0.005	− 0.166~0.177	0.061	0.169
PTI	0.960	0.936~0.975	−0.005	−0.088~0.103	0.034	0.093

*ICC* Intraclass correlation coefficient, *LoA* Limits of agreement, *CI* Confidence interval, *SEM* Standard error of measure, *MDC*_*95*_ Minimal detectable change at 95% confidence level, *deg* degree

### Inter-observer reliability

There was adequate inter-observer reliability for all parameters that described patellar position, trochlear dysplasia, and patellar height (ICCs = 0.821–0.979). The 95% LoA for CA between the two evaluators was slightly wider than those for the other MRI measurements. Table 3 presents the detailed inter-observer reliability.

**Table 3:** Inter-observer reliability of the MRI measurements for patellar instability

Measurement	ICC	95%CI	Mean difference	95%LoA	SEM	MDC_95_
Patellar position						
Angle of Fulkerson, deg	0.975	0.961~0.983	0.275	−1.717~2.266	0.732	2.029
Angle of Grelsamer, deg	0.870	0.814~0.910	−0.157	−4.695~4.381	1.632	4.523
Angle of Laurin, deg	0.931	0.897~0.954	0.480	−3.081~4.042	1.320	3.658
PTA, deg	0.952	0.930~0.968	−0.039	−2.411~2.332	0.854	2.367
LPD, mm	0.979	0.969~0.986	−0.087	−0.916~0.741	0.303	0.840
BSO, %	0.923	0.887~0.948	−0.004	−0.040~0.033	0.013	0.037
CA, deg	0.906	0.864~0.935	−0.392	−11.146~10.362	3.774	10.460
Trochlear dysplasia						
SA, deg	0.963	0.946~0.975	−0.137	−3.919~3.645	1.366	3.786
LTI, deg	0.963	0.939~0.977	−0.392	−2.549~1.765	0.819	2.272
TFA, %	0.822	0.748~0.876	−0.008	−0.146~0.130	0.050	0.138
TGD, mm	0.931	0.900~0.953	0.011	−0.972~0.994	0.353	0.979
Patellar height						
ISI	0.821	0.725~0.886	−0.006	−0.152~0.141	0.052	0.145
MISI	0.879	0.810~0.923	0.005	−0.189~0.199	0.069	0.191
CDI	0.909	0.957~0.943	−0.001	−0.114~0.112	0.040	0.112
BPI	0.885	0.820~0.927	0.010	−0.145~0.165	0.056	0.154
PTI	0.956	0.930~0.973	0.008	−0.088~0.103	0.034	0.095

*ICC* Intraclass correlation coefficient, *LoA* Limits of agreement, *CI* Confidence interval. SEM: standard error of measure, *MDC*_*95*_ Minimal detectable change at 95% confidence level, *deg* degree

### Inter-scan reliability

**Fig. 4:**
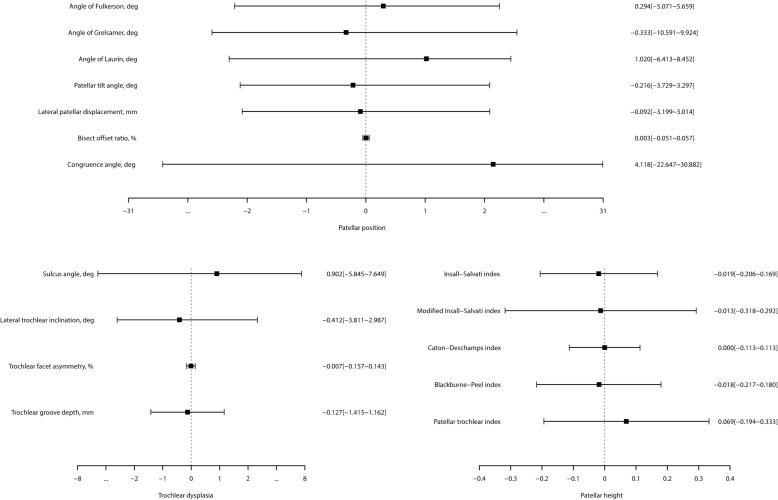
The 95% limits of agreement and the mean differences for inter-scan reliability

The patellar position indices, including the angle of Fulkerson, angle of Laurin, PTA, LPD, and BSO, showed adequate inter-scan reliability with relatively higher ICCs (ICCs = 0.723–0.897) and narrower 95% LoAs. The angle of Grelsamer and CA showed inadequate inter-scan reliability with relatively lower ICCs (ICCs = 0.325–0.380) and wider 95% LoAs. All trochlear dysplasia indices showed adequate inter-scan reliability (ICCs = 0.793–0.915). Nearly all patellar height indices showed adequate inter-scan reliability (ICCs = 0.700–0.903), except for PTI (ICC = 0.655). The detailed inter-scan reliability is shown in Table 4 and Fig. 4.

**Table 4:** Inter-scan reliability of the MRI measurements for patellar instability

Measurement	ICC	95%CI	Mean difference	95%LoA	SEM	MDC_95_
Patellar position						
Angle of Fulkerson, deg	0.836	0.729~0.903	0.294	−5.071~5.659	1.918	5.315
Angle of Grelsamer, deg	0.325	0.054~0.550	−0.333	−10.591~9.924	3.674	10.184
Angle of Laurin, deg	0.723	0.559~0.832	1.020	−6.413~8.452	2.732	7.573
PTA, deg	0.897	0.828~0.940	−0.216	−3.729~3.297	1.262	3.497
LPD, mm	0.726	0.564~0.834	−0.092	−3.199~3.014	1.108	3.072
BSO, %	0.827	0.716~0.897	0.003	−0.051~0.057	0.020	0.054
CA, deg	0.380	0.128~0.588	4.118	−22.647~30.882	9.873	27.367
Trochlear dysplasia						
SA, deg	0.887	0.808~0.934	0.902	−5.845~7.649	2.476	6.864
LTI, deg	0.915	0.855~0.951	−0.412	−3.811~2.987	1.242	3.443
TFA, %	0.793	0.663~0.876	−0.007	−0.157~0.143	0.054	0.149
TGD, mm	0.881	0.801~0.930	−0.127	−1.415~1.162	0.467	1.295
Patellar height						
ISI	0.700	0.481~0.837	−0.019	−0.206~0.169	0.068	0.188
MISI	0.728	0.520~0.854	−0.013	−0.318~0.292	0.108	0.299
CDI	0.903	0.815~0.951	0.000	−0.113~0.113	0.040	0.111
BPI	0.820	0.671~0.906	−0.018	−0.217~0.180	0.071	0.198
PTI	0.655	0.373~0.819	0.069	−0.194~0.333	0.099	0.274

*ICC* Intraclass correlation coefficient, *LoA* Limits of agreement, *CI* Confidence interval. *SEM* Standard error of measure, *MDC*_*95*_ Minimal detectable change at 95% confidence level, *deg* degree

## Discussion

We undertook detailed assessments of the established MRI measurements that are employed to distinguish between patients with PI and normal status, including parameters that describe the patellar position, trochlear dysplasia, and patellar height [9, 14, 21]. In addition, we compared the reliability of these measurements based on two MRI scans from a single person, which was more meaningful in regard to clinical examination. We found that: (1) all measurements showed adequate intra- and inter-observer reliability on MRI; (2) most measurements showed adequate inter-scan reliability, except for the angle of Grelsamer, CA, and PTI.

A recent meta-analysis identified a reasonable level of reliability for measurements of patellar height and sulcus angle on MRI images; however, it showed insufficient evidence to determine the reliability of other measurements [6]. In this study, the measurements showed adequate intra- and inter-observer reliability. Van Huyssteen [22] and Ali [23] illustrated the difference in trochlear dysplasia measurements obtained at different locations. The patella begins to enter the trochlea at 20°–30° knee flexion [24]. Thus, it is very likely that the femoral trochlea will be invisible on the axial slice through the largest axis of the patella, especially at full extension of the knee [25, 26]. The correct selection of the image slice and bony landmark is the key to accurate parameter measurements. The adequate reliability observed in this study may be due to the measurement methods involving superimposition of multiple slices.

Some previous studies have evaluated the reliability of PI-related measurements. Charles et al. [9] found that the ICC value of patellar tilt measurements was greater than 0.9 when the knees were in the non-weight-bearing state in full extension. Becher [10] showed that the general reliability ranged from good to excellent for ISI, CDI, PTI, PTA, and BSO at 0°, 15°, 30°, and 45° flexion, respectively. The reliability of PI measurements was evaluated only in the same knee position in those studies [6, 9, 10]. However, PI measurements were significantly influenced by knee position, such as knee flexion angle [10, 11, 13, 27]. In clinical MRI practice, it is difficult to place the knee in the same position between different examinations due to the shape of the knee coil, the knee location in the coil, and inter-operator differences [12]. The inter-scan reliability in this study was close to that seen in common practice.

In this study, the angle of Fulkerson, PTA, and BSO were more reliable between scans to assess the patellar position. All trochlear dysplasia parameters were reliable between scans. ISI, MISI, BPI, and CDI were reliable between scans to assess the patellar height, with CDI being the most reliable. In addition, previous studies have reported CDI to be reliable for knees of different physical sizes, variable skeletal maturation, patellar pole abnormalities, and after osteotomy of the tibial tubercle [1, 28, 29].

Most measurements showed adequate inter-scan reliability, except for the angle of Grelsamer, CA, and PTI. For the angle of Grelsamer, the poor inter-scan reliability may be associated with the rotation of the knee according to the measurement method. For CA, the inter-scan reliability was the poorest among all of the studied parameters. Marzo et al. [27] reported that the CA was significantly reduced when the weight-bearing knee was flexed at 30° because of the bony constraint by the trochlea on the patella. There was little bony constraint in the patients at 7.25° mean flexion in this study. The poor inter-scan reliability of CA may be attributed to the difficulties in determining the sleek patellar apex in dot form and the subtle changes of patellar position caused by knee repositioning within a scanner. Future research is necessary to determine the effect of small changes in knee flexion on patellar position. PTI could directly reveal the true relationship between the patella and the femur, and was affected by changes in the knee flexion angle [10]. The inadequate inter-scan reliability may be related to changes in the knee flexion angle.

It is likely that all measurements will have some error that originates from the evaluator, operator, patient, and so on. The MDC is an important index to interpret whether there is true change beyond that implied by the measurement error. Few studies on the reliability of PI measurements have been based on MDCs. In this study, various MDC values of PI measurements for inter-scan reliability were studied, and these can be used in future longitudinal studies to identify true change and the measurement error.

There are some limitations of this study. First, the knee flexion angle could not be controlled for a retrospective study. Therefore, the difference in the angle in each pair of images was not same. Second, the 3-mm slice thickness may negatively affect measurement accuracy. Third, some commonly used parameters, such as the tibial tubercle–trochlear groove distance and the varus–valgus angle, were not assessed in this study. Finally, further stratification based on age, sex, BMI, and diagnosis was not undertaken because of the small sample size.

## Conclusion

All measurement methods for assessing PI showed adequate intra- and inter-observer reliability on MRI. Most measurements showed adequate inter-scan reliability, except for the angle of Grelsamer, CA, and PTI.

## Additional file

Additional file 1 : Description of measurements. (PDF 73 kb)

## Data Availability

The datasets analyzed in this study are available from the corresponding author on reasonable request.

## Abbreviations

BPI: Blackburne–Peel index
BSO: Bisect offset ratio
CA: Congruence angle
CDI: Caton–Deschamps index
CI: Confidence interval
ICC: Intraclass correlation coefficient
ISI: Insall–Salvati index
LoA: Limit of agreement
LPD: Lateral patellar displacement
LTI: Lateral trochlear inclination
MISI: Modified Insall–Salvati index
MRI: Magnetic resonance imaging
PI: Patellar instability
PTA: Patellar tilt angle
PTI: Patellar trochlear index
SA: Sulcus angle
TE: Echo time
TFA: Trochlear facet asymmetry
TGD: Trochlear groove depth
TR: Repetition time
