# Supplementary material for: Patellar instability: the reliability of magnetic resonance imaging measurement parameters
Source: BMC Musculoskelet Disord. 2019 Jul 6;20:317. doi: 10.1186/s12891-019-2697-7 (PMC6612413; doi:10.1186/s12891-019-2697-7)
Supplement: Supplementary file 1 — : Description of measurements. (PDF 73 kb) [file 12891_2019_2697_MOESM1_ESM.pdf]

Description of measurements

Measurement	
	Slice I: the axial slice that shows the most posterior aspect of the femoral condyles; Slice II: the axial slice through the largest axis of the patella; Slice III: the first axial slice (going from proximal to distal) that shows cartilage spanning the whole trochlear surface. Reference line A: a tangential line through the posterior femoral condyle on Slice I; Reference line B: a tangential line through the anterior femoral condyle on Slice III.
**Patellar position**	
Angle of Fulkerson, deg [9]	Line C is drawn along the lateral articular facet of the patella on Slice III. The angle between C and A is the patellar angle of Fulkerson.
Angle of Grelsamer, deg [9]	Line D is drawn through the largest transverse axis of the patella on Slice II. The angle between this and the horizontal axis is the patellar angle of Grelsamer.
Angle of Laurin, deg [9]	Line C is drawn tangential to the lateral facet of the patella on Slice II. The angle between C and B is the patellar angle of Laurin.
PTA, deg [9,10,14]	Line D is drawn through the largest transverse axis of the patella on Slice II. The angle between D and A is PTA.
LPD, mm [15]	Line E is drawn perpendicular to A through the medial margin of the patella facet on Slice II, and line F is drawn perpendicular to the A through the highest point of the medial femoral condyle on Slice III. The LPD is the shortest distance between E and F.
BSO, % [10]	A line is drawn perpendicular to A through the deepest point of the trochlear groove on Slice III. The patellar length (P) is divided into the medial (M) and lateral (L) lengths by this on Slice II. BSO=L/P.
CA, deg [9]	Line G is drawn along the bisector of the sulcus angle on Slice III. Line H is drawn from the deepest point of the trochlear groove to the patellar apex on Slice III. The angle between G and H is CA.
**Trochlear dysplasia**	
SA, deg [9,14]	Two lines, M and L, are drawn along the slopes of the medial and lateral trochlea on Slice III respectively, and the angle between them is SA.
LTI, deg [14]	Line I is drawn along the lateral facet of the trochlear groove on Slice III. LTI is the angle between I and A.
TFA, % [14]	Lengths M and L are measured from the medial and lateral margins of the trochlea to the deepest point of the trochlear groove on slice III respectively, representing the lengths of the medial and lateral facets of the trochlea. The TFA is the ratio of length M to length L.
TGD, mm [14,16]	Lengths M, L, and J are measured as the distances of the medial and lateral femoral condyles and the deepest point in the trochlear groove perpendicular to A on Slice III respectively. TGD=(M+L)/2-J.
**Patellar height**	The following measurements are made on the sagittal slice showing the greatest length of the patella: Height A: Measured from the most cranial aspect of the patella to the most caudal aspect of the

	Height B: Measured from most cranial to most caudal margin of the patellar articular surface.
	Height C: Measured from the most caudal aspect of the patella to the most cranial point of insertion of patellar tendon to the tibia, equivalent to patellar tendon length.
	Height D: Measured from the most caudal aspect of the patellar articular surface to the anterior margin of the tibia plateau.
	Height E: Measured from the most caudal aspect of the patellar articular surface to the most cranial point of insertion of patellar tendon to the tibia.
	Height F: the vertical distance from the most caudal aspect of the patellar articular surface to a line drawn along the tibia plateau.
	Height G: The height of the overlap between trochlear and patellar articular cartilage.
ISI [9,10,14]	The ratio of C to A
MISI [9]	The ratio of E to B
CDI [9,10,14]	The ratio of D to B
BPI [13]	The ratio of F to B
PTI [10,14]	The ratio of G to B

PTA: patellar tilt angle; LPD: lateral patellar displacement; CA: congruence angle; BSO: bisect offset ratio; SA: sulcus angle; TGD: trochlear groove depth; LTI: lateral trochlear inclination; TFA: trochlear facet asymmetry ISI: Insall-Salvati index MISI: modified Insall-Salvati index CDI: Caton-Deschamps index BPI: Blackburne-Peel index PTI: patellar trochlear index
